# A rare cause of post-traumatic low back pain: Lumbar Morel-Lavallée lesion in a collegiate athlete — A case report

**DOI:** 10.1016/j.inpm.2026.100828

**Published:** 2026-10-15

**Authors:** Rohin Thomas, Lisa Huynh

**Affiliations:** Department of Orthopaedic Surgery, Stanford University, 450 Broadway St., Pavilion C; 4th Floor, MC 6342, Redwood City, CA, 94063, USA

**Keywords:** Morel-Lavallée lesion, Lumbar spine, Low back pain, Soft-tissue injury, Sports injury

## Abstract

Morel-Lavallée lesions (MLLs) are closed traumatic degloving injuries caused by separation of the subcutaneous tissue from the underlying fascia. Although most commonly described around the greater trochanter and pelvis, lumbar MLLs are rare and may be overlooked in patients presenting with post-traumatic low back pain. We report a 23-year-old collegiate rugby player who developed severe axial low back pain after being lifted and driven onto his back during a game. Initial computed tomography demonstrated no fracture or traumatic malalignment. Persistent severe pain and focal fluctuant swelling over the lumbar spine prompted magnetic resonance imaging five days after injury, which demonstrated a superficial fluid collection between the dorsal subcutaneous fat and lumbar fascia at L4, consistent with an MLL. The patient was referred for consideration of ultrasound-guided aspiration and treated with continuous compression. Interval ultrasound demonstrated only a minimal residual collection without drainable fluid, and aspiration was deferred. His symptoms progressively improved and completely resolved within four weeks. This case highlights lumbar MLL as an uncommon cause of severe post-traumatic low back pain despite reassuring osseous imaging. Early recognition and appropriate soft-tissue imaging can guide management, help avoid unnecessary invasive treatment, and allow selected small acute lesions to resolve successfully with conservative care.

## 1 Introduction

Morel-Lavallée lesions (MLLs) are closed traumatic soft-tissue degloving injuries in which shearing forces separate the dermis and subcutaneous tissue from the underlying fascia [1]. Disruption of the vascular and lymphatic networks creates a potential space that can fill with blood, hemolymphatic fluid, debris, and necrotic fat [1]. MLLs can occur wherever mobile subcutaneous tissue overlies relatively fixed fascia and most commonly occur over the greater trochanter and are associated with pelvic, acetabular, and lower extremity trauma [1], [2].

Clinical presentation can vary and depends on lesion location, size, and chronicity. Patients may develop pain, soft-tissue swelling, fluctuance, ecchymosis, skin hypermobility, or altered cutaneous sensation. The skin overlying the lesion may appear normal with subtle swelling, and symptoms may not become immediately apparent after the inciting trauma [1]. Failure to recognize the lesion can lead to persistent inflammation, accumulation of hemolymphatic fluid, and eventual formation of a fibrous pseudocapsule, increasing the likelihood of persistent pain, infection, tissue necrosis, and the need for surgical treatment [3].

Lumbar MLLs remain particularly uncommon. The literature mainly consists of individual case reports and small case series that frequently involve high-energy mechanisms, polytrauma, or concomitant spinal and pelvic injuries [4], [5], [6]. The lumbar location of an MLL presents a diagnostic challenge; post-traumatic low back pain is common, early symptoms may resemble paraspinal soft-tissue injury, and initial trauma imaging may focus on osseous or neurologic injury rather than a fascial-plane injury. Falsely reassuring initial trauma imaging and subtle physical findings may lead to delayed recognition of lumbar MLLs.

For clinicians who evaluate post-traumatic low back pain, particularly in spine, sports medicine, and interventional pain settings, recognition of MLLs is important because management differs considerably from mechanical low back pain or uncomplicated soft-tissue injury. We present a lumbar MLL in a collegiate rugby athlete with persistent severe low back pain despite negative computed tomography (CT) imaging that was subsequently identified on magnetic resonance imaging (MRI) and managed with tissue-preserving, conservative management.

## 2 Case

Informed consent was obtained from the patient for publication of this case report and the accompanying clinical images.

A 23-year-old male collegiate rugby player presented with acute post-traumatic low back pain after being lifted and driven onto his back during a game. He was evaluated in the emergency department on the day of injury; a lumbar spine CT showed no fracture or traumatic malalignment. The patient was discharged from the emergency department with conservative treatment.

Three days later, he was evaluated in the spine clinic for persistent, severe low back pain. He described the pain as sharp, 9/10 at its worst, and exacerbated by prolonged sitting, standing, long strides, and direct pressure over the lumbar spine. He denied radicular symptoms, bowel or bladder dysfunction, and saddle sensory changes. Neurologic examination was reassuring, with normal lower-extremity strength, sensation, and reflexes. There was focal midline fluctuant swelling and exquisite tenderness at L3–L4 without overlying ecchymosis or palpable step-off. The patient was instructed to continue conservative treatment, and a lumbar MRI was ordered to evaluate for occult osseous or ligamentous injury not identified on previous CT. MRI obtained 5 days after the injury demonstrated a superficial fluid collection between the dorsal subcutaneous fat and underlying lumbar fascia at L4, consistent with a Morel-Lavallée lesion (Fig. 1).

**Fig. 1:**
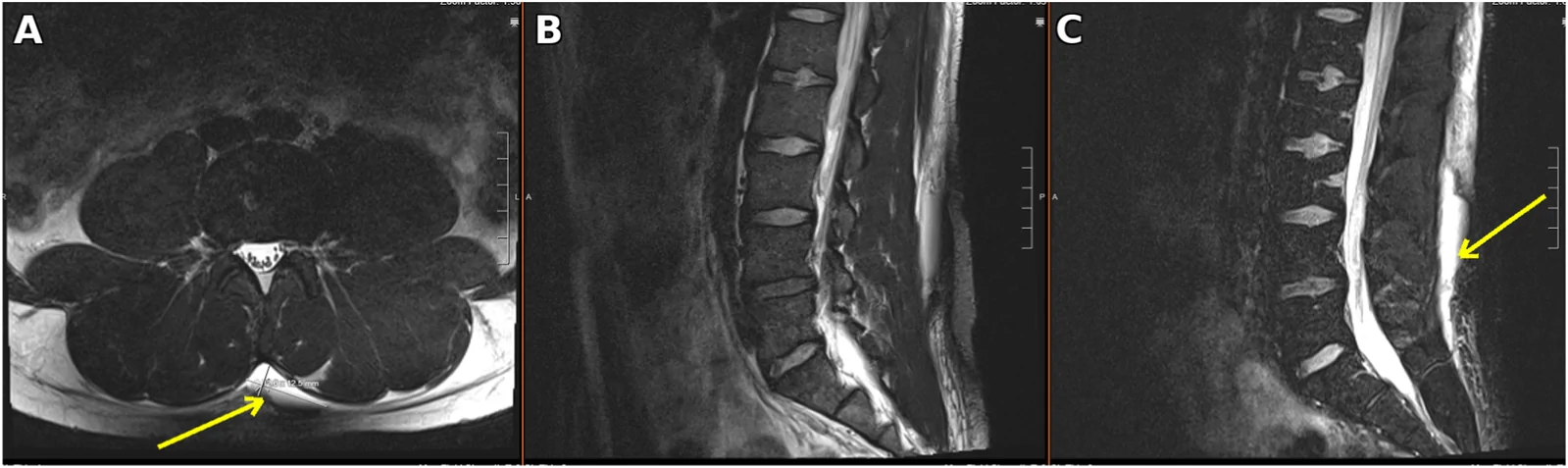
Lumbar spine MRI obtained five days after injury. **(A)** Axial T2-weighted image demonstrating a superficial fluid collection in the posterior subcutaneous plane at L3–L4 (arrow). **(B)** Sagittal T2-weighted image. **(C)** Sagittal STIR image showing hyperintense fluid signal at the fat–fascia interface of the posterior soft tissues (arrow), consistent with a Morel-Lavallée lesion.

He was referred to sports medicine for consideration of ultrasound-guided aspiration and was advised to apply continuous compression. At evaluation six days after MRI, diagnostic ultrasound demonstrated only a minimal residual anechoic collection without a drainable fluid collection; therefore, aspiration was deferred. Given the interval clinical and sonographic improvement, conservative management was continued. His pain progressively improved over the subsequent 2 weeks, with complete resolution by 4 weeks.

## 3 Discussion

This clinical case illustrates an uncommon location of a Morel-Lavallée lesion in an athlete with severe post-traumatic axial low back pain. The clinical value of the case is not simply the unusual location. Rather, the case demonstrates a diagnostic sequence relevant to spine and pain clinicians. The patient's initial CT excluded fracture and traumatic malalignment but did not explain his severe persistent symptoms. Recognition of the injury ultimately depended on integrating the traumatic shearing mechanism with the focal finding of fluctuant swelling over the lumbar spine and obtaining MRI to evaluate the soft tissues. Ultimately, diagnostic ultrasound was used to determine whether planned percutaneous intervention remained necessary.

### 3.1 Pathophysiology and clinical presentation

The defining injury in an MLL is traumatic separation of the subcutaneous tissue from the underlying deep fascia. Tangential or shearing forces applied to the mobile subcutaneous tissues across the relatively fixed fascia disrupt perforating capillaries and lymphatic vessels. The resulting potential space may fill with blood, lymph, fat, and necrotic debris [1]. As blood products organize and inflammatory changes develop, the composition and imaging appearance of the collection subsequently evolve [7].

MLLs most commonly occur where the skin and subcutaneous tissues are highly mobile relative to the underlying fascia, typically around the greater trochanter and proximal thigh [1]. The lumbar region is a less common location [4], [5]. However, the same biomechanical and pathophysiological principles apply in our case. Being lifted and driven onto the back during rugby likely generated a combination of direct impact and shear across the posterior lumbar soft tissues. Although the direction and magnitude of the tissue-plane forces cannot be determined, the mechanism was compatible with the subsequent localization of the collection at the dorsal fat–fascia interface.

The clinical appearance of MLLs can range from obvious soft-tissue deformity and ecchymosis to more subtle findings. Pain, swelling, fluctuance, compressibility, skin hypermobility, and altered cutaneous sensation have all been described [1]. Symptoms may develop immediately or be delayed, leading to missed diagnoses and the possibility of more invasive treatment [1], [3]. In this patient with negative fracture imaging on CT, absence of ecchymosis or substantial visible trauma made the palpable midline fluctuance particularly important. The combination of severe focal tenderness and a fluctuant collection following a shearing injury provided the clinical clue that the patient's symptoms represented more than an uncomplicated lumbar contusion.

### 3.2 Lumbar Morel-Lavallée lesions and the differential diagnosis of post-traumatic low back pain

Lumbar MLLs have been described less frequently than lesions of the pelvis and lower extremities. Prior reports include cases in adolescents, patients involved in motor vehicle collisions, patients with polytrauma, and patients with associated thoracolumbar or pelvic fractures [4], [5], [6], [8]. Sawkar et al. described two lumbar MLLs in adolescents following motor vehicle accidents (MVA), while other authors reported acute lumbar lesions following blunt trauma [4], [8]. Mulcahy and Ball reported lumbar MLLs occurring in association with thoracolumbar fractures, emphasizing that the lesions may be overlooked during the initial assessment of patients with more obvious spinal injuries [6]. More recent reports have similarly emphasized the potential for delayed recognition, persistent symptoms, and progression to more complex lesions requiring procedural or surgical management [4], [5], [9].

This case differs from the previously reported lumbar MLLs: the patient was a collegiate athlete with an isolated lumbar soft-tissue injury, no associated fracture or polytrauma, and no neurologic deficit. His primary manifestation was severe axial low back pain rather than the competing injuries commonly encountered in a polytrauma population. This presentation is relevant to outpatient spine and pain practices, where patients may be evaluated after initial emergency imaging has already excluded fracture.

The differential diagnosis of post-traumatic low back pain is broad and includes vertebral fracture, ligamentous injury, paraspinal muscle injury, and other soft-tissue trauma. In the setting of a focal fluid collection, the differential expands to include hematoma, seroma, fat necrosis, abscess, and, in delayed presentations, soft-tissue neoplasms. Consider an MLL when a traumatic shearing mechanism is accompanied by focal swelling or fluctuance over a fascial plane.

### 3.3 Role of imaging

Imaging is essential to confirm MLLs and identify the potential space between the subcutaneous tissue and fascial layer. Often, CT is the first cross-sectional study obtained after trauma. It is primarily performed to evaluate osseous injury or malalignment, and early MLLs may be subtle or missed [1]. MRI is the preferred modality for characterizing MLLs because it provides better soft-tissue contrast and can define the location, size, composition, and presence of a capsule [1], [7]. Typical MRI findings include a fusiform or laminar collection between the subcutaneous fat and deep fascia, with signal characteristics that vary by the collection's age and composition [7].

The Mellado and Bencardino classification describes six MRI patterns of Morel-Lavallée lesions: type I, seroma; type II, subacute hematoma; type III, chronic organizing hematoma; type IV, perifascial dissection with closed fatty laceration; type V, pseudonodular lesion; and type VI, infected lesion. The types are distinguished by lesion morphology, signal characteristics, capsule formation, enhancement, and evidence of infection [7]. Typically, type I seroma-like collections are T1 isointense and T2 hyperintense, as seen in our patient's MRI findings, although a formal Mellado-Bencardino classification was not assigned in the radiology report. MRI findings can help describe morphology but do not dictate treatment [3], [10].

Ultrasound is a complementary imaging tool; it can confirm fluid collection, assess residual volume, distinguish a persistent collection from edematous tissue, guide aspiration when intervention is appropriate, and is suited for interval assessment [10].

### 3.4 Management

There is no universally accepted treatment algorithm for MLLs, given their variation in presentation. Treatment options include observation and compression, percutaneous aspiration or drainage, sclerodesis, and open debridement with elimination of the residual dead space. Small acute lesions without a mature capsule may respond to compression and close clinical observation. Compression may reduce the potential space between the tissue planes and may help facilitate fluid reabsorption. This conservative approach is appropriate if overlying tissue is viable, the collection is small, and the patient can be followed for resolution or recurrence [3], [10].

Percutaneous aspiration can be considered when a symptomatic collection is large and accessible. However, recurrence is possible, especially in larger lesions, when a capsule persists, or when potential space remains [10]. In the Mayo Clinic series, recurrence was much more common with aspirations more than 50 mL, leading authors to recommend operative intervention when aspiration exceeds the 50 mL threshold [11]. Persistent or recurrent lesions may be treated with drainage combined with doxycycline sclerodesis [12].

Chronic lesions may cause persistent inflammation, leading to formation of a fibrous pseudocapsule that prevents adherence of the separated tissue planes, promoting recurrent fluid accumulation. Compression and aspiration are less likely to provide durable resolution once this capsule forms and treatment becomes more invasive. Surgical strategies include debridement, capsulectomy, quilting sutures, or drains to help eliminate the dead space [3], [10]. Infection, skin necrosis, recurrent collection, and proximity to planned operative approaches can further lower the threshold for surgical management [3], [6], [10].

Our patient's course demonstrates successful conservative treatment of an acute lumbar Morel-Lavallée lesion. The lesion was identified within the first week after injury on MRI, and interval ultrasound demonstrated spontaneous reduction of the collection, allowing planned aspiration to be deferred. Continued compression was followed by complete symptom resolution within 4 weeks. The favorable outcome likely reflects early recognition of a relatively small lesion before development of a chronic pseudocapsule or other complication. This outcome should not be generalized to larger, recurrent, encapsulated, infected, or progressive lesions, which may require procedural or surgical treatment [3], [10].

Although aspiration or surgery was ultimately unnecessary, early diagnosis remained clinically meaningful. It explained the patient's severe pain despite negative fracture imaging, allowed targeted compression and surveillance, and prompted appropriate referral for consideration of drainage. Delayed or missed MLLs may become persistent, recurrent, infected, or symptomatic as pseudocapsule formation occurs [3], [10]. Mulcahy and Ball reported substantial wound complications in patients with MLLs accompanying thoracolumbar fractures, highlighting the potential morbidity of unrecognized lesions, particularly when surgical approaches traverse compromised tissue planes [6].

This case also has practical implications for interventional pain, spine, and sports medicine physicians. Severe post-traumatic axial low back pain with negative fracture imaging may prompt consideration of common spinal pain generators; however, not all pain localized to the lumbar region originates from the vertebral column, discs, facets, sacroiliac joints, or paraspinal musculature. A Morel-Lavallée lesion represents a superficial fascial-plane injury and therefore requires a distinct diagnostic and treatment pathway. Recognition of the lesion before proceeding with an unrelated spinal intervention may prevent treatment directed at the wrong pain generator. The interventionalist's role is therefore not simply to perform a technically feasible procedure, but to integrate clinical examination and imaging findings to determine whether an intervention is indicated.

Lumbar Morel-Lavallée lesions should therefore remain in the differential diagnosis after direct or tangential trauma when patients present with focal edema, tenderness, or fluctuance, particularly when the severity of axial low back pain is discordant with otherwise reassuring osseous imaging. Because lumbar MLLs are uncommon and easily overlooked, careful inspection and palpation of the posterior soft tissues may provide a diagnostic clue not apparent on neurologic examination or routine fracture evaluation.

## 4 Conclusion

Lumbar Morel-Lavallée lesions are an uncommon but clinically important cause of post-traumatic low back pain. Negative fracture imaging should not provide false reassurance when severe focal pain and fluctuant swelling persist after a shearing injury. MRI can establish the diagnosis and characterize the lesion, while ultrasound may provide interval assessment and guide procedural decision-making. Small acute lesions may resolve with compression and observation, as demonstrated in this collegiate athlete. Early recognition can facilitate appropriate management, avoid unnecessary intervention, and potentially prevent progression to a chronic encapsulated lesion requiring more invasive treatment.
